# Age-Dependent Positivity-Bias in Children’s Processing of Emotion Terms

**DOI:** 10.3389/fpsyg.2017.01268

**Published:** 2017-07-26

**Authors:** Daniela Bahn, Michael Vesker, José C. García Alanis, Gudrun Schwarzer, Christina Kauschke

**Affiliations:** ^1^Clinical Linguistics, Department of German Linguistics, Philipps University of Marburg Marburg, Germany; ^2^Department of Developmental Psychology, Justus Liebig Universität Gießen Gießen, Germany; ^3^Neuropsychology Section, Experimental and Biological Psychology, Department of Psychology, Philipps University of Marburg Marburg, Germany

**Keywords:** lexical decision, emotional categorization, valence decision, emotion terms, children

## Abstract

Emotions play an important role in human communication, and the daily-life interactions of young children often include situations that require the verbalization of emotional states with verbal means, e.g., with emotion terms. Through them, one can express own emotional states and those of others. Thus, the acquisition of emotion terms allows children to participate more intensively in social contexts – a basic requirement for learning new words and for elaborating socio-emotional skills. However, little is known about how children acquire and process this specific word category, which is positioned between concrete and abstract words. In particular, the influence of valence on emotion word processing during childhood has not been sufficiently investigated. Previous research points to an advantage of positive words over negative and neutral words in word processing. While previous studies found valence effects to be influenced by factors such as arousal, frequency, concreteness, and task, it is still unclear if and how valence effects are also modified by age. The present study compares the performance of children aged from 5 to 12 years and adults in two experimental tasks: lexical decision (word or pseudoword) and emotional categorization (positive or negative). Stimuli consisted of 48 German emotion terms (24 positive and 24 negative) matched for arousal, concreteness, age of acquisition, word class, word length, morphological complexity, frequency, and neighborhood density. Results from both tasks reveal two developmental trends: First, with increasing age children responded faster and more correctly, suggesting that emotion vocabulary gradually becomes more stable and differentiated during middle childhood. Second, the influence of valence varied with age: younger children (5- and 6-year-olds) showed significantly higher performance levels for positive emotion terms compared to negative emotion terms, whereas older children and adults did not. This age-related valence effect in emotion word processing will be discussed with respect to linguistic and methodological aspects.

## Introduction

The ability to verbalize emotional states is a crucial stepping stone not only in language acquisition, but also for a child’s social-emotional development, since it enables children to participate in social contexts which form an essential learning environment. In their third year of life children begin to produce their first words to express emotional states (such as *happiness*) or emotional behavior (e.g., *crying*, Bretherton and Beeghly, 1982; Kristen et al., 2012). For this purpose, they gradually acquire various linguistic devices, including, most notably, emotion terms. Emotion terms are lexical items such as *fear*, which directly refer to emotions as symbols (Schwarz-Friesel, 2013). The emotive vocabulary constantly grows in the course of language development, and is not fully elaborated until early adolescence (Baron-Cohen et al., 2010). In contrast to emotion terms, so-called affective words trigger an affective reaction because their meaning includes an emotional connotation (e.g., *death*), but they do not explicitly denote an emotional state. While affective words can either be concrete (e.g., *bomb*) or abstract (e.g., *violence*), Vigliocco et al. (2013) suggest that emotion terms form a bridge between those two categories, since they contain both abstract and concrete pieces of information, and should therefore be treated as an independent word category between abstract and concrete words. Inherent semantic features of emotion terms are (1) that they are emotional (as opposed to neutral) and (2) that they are characterized by a specific valence (positive or negative) and by a word-specific degree of arousal (low or high).

Valence, the perceived value of a stimulus’ pleasantness, was shown to be one of two basic dimensions along which humans classify emotional content from their environment (Russell and Ridgeway, 1983). Infant studies in face perception revealed that even newborns can distinguish facial expressions along their valence (Farroni et al., 2007), reflecting that the ability to categorize new information by its valence is highly relevant in human behavior, e.g., for evolutionary adaptive functions. The impact of emotionality and valence on word processing was demonstrated by many studies with adults as expressed by a processing advantage for emotionally toned over neutral ones, as well as by an advantage of positive over negative words or vice versa. In light of the fundamental importance for human behavior to categorize perceived information as positive or negative, it is surprising that so far little is known about how valence and emotionality impact children’s word processing.

The present study aims to shed light on the question of if and how valence influences children’s processing of emotion terms. Can an enhanced processing of positive or negative emotional content in children be observed in language, and do changes occur in the course of development? Answering these questions will contribute to a comprehensive understanding of the development of emotion processing skills during childhood. As stated above, emotion terms are characterized by carrying a specific value of valence. Moreover, their acquisition starts in early childhood (Kauschke and Klann-Delius, 1997) which makes them serve as an eminently suitable word category in studying the categorization abilities of children from different age groups with respect to the valence dimension.

When investigating emotion and valence effects researchers often use the well-known psycholinguistic lexical decision paradigm (lexical decision task, LDT) in which visually or verbally presented letter or phoneme strings are to be judged as a word or a pseudoword (Goldinger, 1996). The processing of positive and negative word stimuli can also be assessed with emotional categorization tasks (often referred to as valence identification tasks or valence decision tasks, VDT), where participants are asked to categorize stimuli along their value of pleasantness. Hereafter, we will first describe existing findings for emotion and valence effects in adults’ word processing, and then present the much smaller number of similar studies in children.

It is generally agreed that affective features of a word’s meaning (such as emotionality, characterized by a certain value of valence and arousal) influence performance levels in word processing. A large number of studies using LDT point toward a processing advantage of emotionally toned words over neutral ones (for English: Scott et al., 2009; Citron et al., 2013; Yap and Seow, 2014; Ponari et al., 2015; Goh et al., 2016, for German: Kanske and Kotz, 2007; Hofmann et al., 2009; Kousta et al., 2009; Palazova et al., 2011). Consistently, faster response times and higher accuracy rates are reported for visually presented positive and negative words compared to neutral words. These results substantiate the existence of an emotion effect in word processing, and suggest that it holds across languages. Vinson et al. (2014) explain the facilitating effect of emotionality in terms of grounding word meanings in emotional experience. Another cause of this effect, as stated by Kousta et al. (2009), might be the greater motivational relevance of emotional stimuli over neutral ones, since processing positive and negative information is highly important for survival. Despite the fact that the underlying mechanisms of the emotion effect are not yet fully understood, it is obvious that emotional systems are involved in single word processing.

Research with adult participants shows heterogeneous results regarding the question of whether negative or positive words are processed more efficiently. A negativity bias, i.e., an improved perception of negative words in the form of faster responses and/or higher accuracy rates, was found in several studies using VDTs (e.g., Siegle et al., 2002; Dijksterhuis and Aarts, 2003; Estes and Verges, 2008; Nasrallah and Carmel, 2009). Participants showed faster and more correct responses in categorizing negative words by their valence and were therefore more competent in accessing valence information from negative words compared to positive words. In addition, Palazova et al. (2013) demonstrated preferential processing of negative words in a LDT. The negativity bias has been explained in terms of an automatic vigilance for negative stimuli (Pratto and John, 1991). According to this theory, automatic stimulus evaluation is a mechanism to direct attention toward possibly life-threatening events. Therefore it was assumed that the human brain is more focussed on filtering and analyzing negative information from the environment in order to avoid undesirable consequences.

However, the majority of studies with adults using VDT, LDT, and other experimental approaches such as memory, attention, reading, or naming tasks (e.g., Inaba et al., 2005; Peréz-Edgar and Fox, 2007; Herbert et al., 2008; Kissler et al., 2009; Hinojosa et al., 2010) provide evidence for a positivity bias observed across several languages (e.g., for English, Chinese, Japanese, Spanish, and German). Studies using the VDT with positive and negative affective words (e.g., Võ et al., 2006; Goh et al., 2016) and emotion terms (Feyereisen et al., 1986) report faster reactions when participants were asked to categorize positive word stimuli compared to negative ones. LDTs also found evidence for enhanced word processing in positive words: Participants were faster at identifying positive compared to negative words (Kuchinke et al., 2005, 2007; Kanske and Kotz, 2007; Hofmann et al., 2009; Scott et al., 2009, 2014; Palazova et al., 2011; Bayer and Schacht, 2014; Ferré and Sanchez-Casas, 2014; Kuperman et al., 2014; Ponari et al., 2015; Yao et al., 2016). Kuchinke et al. (2007), Estes and Adelman (2008), Hofmann et al. (2009), as well as Bayer and Schacht (2014) additionally found a positivity advantage for accuracy rates in LDT.

As mentioned above, all previously reported studies investigating the effect of emotionality and valence were conducted with adult participants using written word stimuli. Studies on affective and emotion word processing in children are sparse. One study by Sylvester et al. (2016) used a VDT to shed light on the role of valence in children’s word perception. The 47 pupils (Austrian speakers) between 9 and 12 years of age were asked to categorize 90 affective words as quickly and as accurately as possible by their valence (positive, or negative, or neutral). The 90 words were taken from the kidBAWL (Jacobs et al., 2015), which is a subset of the Berlin Affective Word List, a database including 3000 German words (Võ et al., 2006, 2009). The kidBAWL-items were selected with respect to their usability in developmental studies on language and reading acquisition. Besides a general decrease of response times with increasing age, Sylvester et al. (2016) found evidence for a positivity superiority effect: reactions to positive words were significantly faster than those to negative and neutral words. Thus, children showed the same behavior as adult participants in a previous study (Võ et al., 2006).

Sylvester et al. (2016) explain their results in terms of Unkelbach et al.’s (2008, 2010) informational density hypothesis, which posits that positive information is processed faster because it is more elaborated and densely clustered, i.e., interconnected in memory compared to negative information. As a reason for why positive words are considered to be more densely clustered Unkelbach et al. (2008, pp. 37–38) suggest that, from a psycholinguistic perspective: “language is more diversified on the negative side, whereas the positive side is more alike and represents the normal state of the world.” For example, it is more likely that a happy, calm, and nice (thus positive) person is also described as warm and friendly, while a nervous and mean person is not automatically perceived as angry or cold. The authors’ assumption is supported by the results of their similarity rating. Participants were asked to judge the similarity of presented word pairs. A cluster analysis revealed that positive word pairs were overall rated as more similar and therefore assumed to be of a higher density than the negative words. A similar result was found by Sylvester et al. (2016): a hierarchical cluster analysis for children’s ratings of words from the kidBAWL showed that the average valence value for positive words clustered more tightly than for negative and neutral words. Further evidence for a higher interconnectivity of positive words can be seen in the results by Hofmann and Jacobs (2014), who found a relationship between semantic cohesion and valence for positive and negative words from the BAWL: The more positive a word is, the higher is its number of semantically associated words. Thus, in word processing tasks a positive word will activate a denser semantic network, i.e., more associated words. Due to spreading activation, these words build a joint positive association based on the shared information of a positive valence which enlarges the chance that the semantic feature “positive” can be retrieved more quickly. In contrast, the lower interconnectivity of negative words might lead to a less extensive activation of semantic neighbors in the mental lexicon. The joint negative association of affected words would thus be weaker slowing down the categorization process (Kuchinke et al., 2005; Unkelbach et al., 2008).

Another more indirect task to explore the valence effect was used by Peréz-Edgar and Fox (2007), who conducted an auditory attention task with 65 seven-year-old children. Participants heard 180 words (60 positive, neutral, and negative words), and were asked to indicate whether they were spoken by a male or female person. The children performed faster for positive words compared to neutral and negative words.

On the other hand, a positivity advantage has not been consistently found in children. Silk et al. (2009) analyzed VDT reactions from 64 pre/early and mid/late pubertal children and adolescents (aged from 8 to 17 years), but found neither any valence-related differences, nor an interaction of valence and age in word processing: children of all age groups processed positive words similarly to negative ones. An explicit advantage for negative stimuli has also been proposed for children. Findings in favor of a negativity bias, however, are mostly based on children’s early non-linguistic behavior. According to Vaish et al. (2008), social referencing behavior of infants strongly suggests that early in life children attend more to and are more influenced by negative rather than positive facets of their environment. The authors also refer to findings that indicate a negativity bias in children’s discourse and in memories about emotionally valenced events. Thus, young children recognize and process positive as well as negative emotional information in order to appropriately adopt their behavior to environmental circumstances. Initially, they seem to weight negative information more strongly than neutral and positive cues for these adaptive processes. In contrast to a large body of research based on non-linguistic evidence, an advantage for negative stimuli has not been shown in the domain of word processing yet.

Several factors have been shown to modulate the role of valence in word processing: word concreteness, arousal, word frequency, and the task itself. To begin with concreteness, Kanske and Kotz (2007) demonstrated an influence of a word’s concreteness on the existence of a valence effect: Responses for positive words in LDTs were only faster when the words were of a high level of concreteness, whereas no effect of valence was detected for abstract positive and negative words. However, almost all previous studies that have investigated the influence of valence on word processing used affective words (that can either be concrete or abstract) or a mixture of affective words and emotion terms as stimuli. Thus, the factor concreteness has not always been sufficiently controlled for so far. Another factor that modulates the processing of positive and negative word stimuli is arousal. Thomas and LaBar (2005) found stronger priming effects for highly arousing negative words, compared to low-arousal negative and neutral words, suggesting that a high value of arousal enhanced word processing. In line with this, Larsen et al. (2008) reported faster responses for negative words with a high value of arousal in a LDT, while a low value of arousal slowed down the processing speed of negative words. The influence of frequency on valence effects was investigated by Scott et al. (2009, 2014). The authors measured faster response times for positive words only when they were highly frequent. Finally, Estes and Verges (2008) suggest the appearance of a valence effect in word perception to be task-dependent. Participants completed a LDT and VDT with 40 positive and 40 negative words. Whereas a positivity bias was found in the LDT, the response times in the VDT showed the opposite pattern, an advantage for negative words.

In summary, many studies with adults have consistently reported an enhanced processing of emotional over neutral word stimuli, while evidence for valence effects is more inconsistent. Although the majority of study results point to a positivity advantage (shown in many studies with adults and in two studies with children), some studies either found no differences between the two valence groups or even the opposite pattern (a negativity advantage). Hence, the direction of the valence effect still remains unresolved. In addition, the factors leading to an enhanced processing (e.g., faster responses or higher accuracy rates) of positive or of negative word stimuli (such as concreteness, arousal, frequency, and task) have not always been sufficiently controlled for in previous research. Most importantly, there is a considerable imbalance with respect to the participant groups investigated so far: the majority of findings is based on adults’ behavior, whereas only three studies investigated the effect of valence in children. To our knowledge, children’s and adults’ processing of affective words or emotion terms have never been compared so far. Thus, it remains unclear whether and how valence effects are modified by age, as developmental studies on emotion and valence effects are extremely sparse.

In light of the reported lack of developmental studies, we employed two experimental reaction time tasks in order to investigate how accurately and how quickly children (from 5 to 12 years of age) and adults process verbally presented German emotion terms: (1) a LDT with neutral words as well as positive and negative emotion terms and (2) a VDT with positive and negative emotion terms. We used emotion terms exclusively, in order to avoid a mixture of semantically different words (emotion terms vs. affective words). Since all participants performed both tasks the experimental design additionally allows for conclusions about the expected valence effect with respect to the task relevancy factor, i.e., whether the stimulus’ valence is important for the optimal solution of the task or not. For the LDT mentally stored lexical units need to be accessed and compared with a presented string of phonemes for checking its existence as a proper word. Meanwhile, for the VDT, semantic information regarding the word’s valence must be accessed from the mental representation of the emotion term. Thus, in VDT valence is clearly task-relevant, whereas in LDT it is not. The order of the two experiments was kept constant for all participants, to maintain their level of motivation. The longer (and potentially more complex) LDT was always conducted first. Error rates and response times for LDT and VDT were analyzed to detect effects of valence and age. Based on the reported findings from children and adults, we put forward the following questions and hypotheses:

(1) Developmental changes in task performance:
  In line with previous word processing studies with children (e.g., Silk et al., 2009; Kauschke et al., 2012), we expect an age-dependent improvement of word processing skills, reflected in higher accuracy and lower reaction times with increasing age in both tasks. Beyond this expectable age-related improvement we aim at investigating whether developmental changes occur in a linear or in a graded, stepwise manner during childhood.
(2) Valence effect:
  (a) Can a valence effect be observed in LDT and VDT? Given that the majority of findings are in favor of a positivity advantage, valence effects were expected in both tasks, with positive emotion terms being processed faster and more accurately than negative emotion terms. Based on the task relevancy factor, we expected valence effects in VDT to be stronger compared to the LDT.
  (b) Does age influence the appearance and direction of this effect? Since developmental studies comparing adults’ and children’s performance in the processing of affective words and emotion terms are as yet unavailable, we cannot derive task-specific directional hypotheses concerning the question of whether, and if so, how, age impacts the expected valence effects.

## Experiment 1 – Lexical Decision

### Participants

Participants were 96 typically developing children of four age groups recruited from local daycare services and schools in the cities of Gießen, Marburg, and surrounding areas (Hesse, Germany): 5-year-olds (*N* = 24, *M* = 5;7, *SD* = 0;2, range = 5;2 to 5;10), 6-year-olds (*N* = 24, *M* = 6;6, *SD* = 0;3, range = 6;0 to 6;11), 9-year-olds (*N* = 24, *M* = 9;5, *SD* = 0;3, range = 9;0 to 9;11), and 12-year-olds (*N* = 24, *M* = 12;3, *SD* = 0;3, range = 12;0 to 12;11). A standardized expressive vocabulary test (for 5-year-olds: AWST-R by Kiese-Himmel, 2005, for older children: WWT by Glück, 2011) ensured age-appropriate lexical skills. Cognitive development was measured with a test of non-verbal intelligence (CPM by Bulheller and Häcker, 2001). Exclusion criteria were a *t*-value below 40 in one or both of the tests. Furthermore, participants with an average accuracy of less than 60% were excluded from the analysis. This amounted to 10% of all participants (nine children from the 5-year-olds and three children from the 6-year-olds).

Twenty-four adult participants (students from the University of Gießen) served as a control group (*M* = 24;6, *SD* = 5;3, range = 18;0 to 44;0).

All participants grew up as monolingual native speakers of German. Gender was balanced both within and between the different age groups (12 female and 12 male participants per group).

### Stimuli

The stimuli consisted of 24 positive (e.g., *hoffen*, Eng. *to hope*) and 24 negative (*leiden*, Eng. *to suffer*) German emotion terms, 48 neutral German words, e.g., *tragen*, Eng. *to carry*, as well as 96 pseudowords, e.g., *bebeien*. Each emotion term set comprised 10 nouns, 9 verbs, and 5 adjectives. The neutral word set included 20 nouns, 18 verbs, and 10 adjectives. The aim of the stimulus construction procedure was to create word sets that differ only with respect to valence, but are controlled for other (psycho-)linguistic features that have been shown to influence word processing: frequency, concreteness, age of acquisition, word class, word length (number of phonemes), morphological complexity (number of morphemes), neighborhood density (number of phonological neighbors, i.e., words that differ from the target word by a single phoneme), and arousal. **Tables 1A,B** give an overview of the stimuli characteristics.

**Table 1A:** Descriptive statistics for selected neutral words, as well as positive, and negative emotion terms controlled for arousal (on a scale from 1 = low-arousing to 5 = high arousal), valence (on a scale from 1 = very negative, over 4 = neutral, to 7 = very positive), concreteness (on a scale from 1 = very abstract, over 4 = neutral, to 7 = very concrete).

Variables	Valence Children rating by Bahn et al. (under review)	Valence Adults emotion terms: rating by Bahn et al. (under review) neutral words: BAWL-R	Arousal Children rating by Bahn et al. (under review)	Arousal Adults emotion terms: rating by Bahn et al. (under review) neutral words: BAWL-R	Concreteness online-rating with adults, unpublished data, obtained by Bahn and colleagues
(1) Positive emotion terms	5.87	5.65	2.88	3.15	3.72
(2) Negative emotion terms	2.43	2.40	2.99	3.36	3.78
(3) Neutral words	Children rated only positive (1) and negative (2) words	3.89	Children rated only positive (1) and negative (2) words	2.06	5.42
One-way ANOVA on each factor with *post hoc* comparisons (Tukey-HSD)	*F*(1) = 416.12, *p* = 0.000	*F*(2) = 262.55, *p* = 0.000 1 vs. 2: *p* = 0.000 1 vs. 3: *p* = 0.000 2 vs. 3: *p* = 0.000	*F*(1) = 1.01, *p* = 0.321	*F*(2) = 72.75, *p* = 0.000 1 vs. 2: *p* = 0.136 1 vs. 3: *p* = 0.000 2 vs. 3: *p* = 0.000	*F*(2) = 51.44, *p* = 0.000 1 vs. 2: *p* = 0.975 1 vs. 3: *p* = 0.000 2 vs. 3: *p* = 0.000

**Table 1B:** Descriptive statistics for selected neutral words, as well as positive, and negative emotion terms controlled for age of acquisition, frequency, number of phonemes and morphemes, neighborhood density, mean duration and mean pitch of recorded word stimuli.

Variables	Age of acquisition (age; months) Online-rating with adults, unpublished data, obtained by Bahn and colleagues	Absolute Frequency 1/Mio ChildLex	Number of phonemes BAWL-R	Number of morphemes BAWL-R	Neighborhood density BAWL-R	Mean duration of recorded word stimuli in seconds	Mean pitch of recorded word stimuli in Hz
(1) Positive emotion terms	4;7	62.49	5.38	1.71	7.54	0.76	153
(2) Negative emotion terms	5;1	40.74	5.25	1.71	7.21	0.74	149
(3) Neutral words	4;5	36.71	5.21	1.71	10.79	0.72	148
One-way ANOVA on each factor	*F*(2) = 1.37, *p* = 0.258	*F*(2) = 1.29, *p* = 0.281	*F*(2) = 0.13, *p* = 0.881	*F*(2) = 0, *p* = 1	*F*(2) = 2.78, *p* = 0.067	*F*(2) = 1.00, *p* = 0.371	*F*(2) = 1.53, *p* = 0.221

The stimuli were originally selected from the BAWL-R (Võ et al., 2009), which is a database of 3000 German words (emotion terms, affective words, and neutral words). It offers values for valence and arousal (rated by 200 adults) as well as norms for several linguistic variables. Based on the valence and arousal norms given in the BAWL-R, candidates for our three word sets (positive, negative, and neutral words) were selected and preliminarily matched for their averaged value of valence and arousal. Candidates for positive words had to show a very high (positive) mean value of valence and a high value of arousal, negative word candidates had to show a very high negative valence value, but also a high value of arousal. Neutral words had to show a valence value around 4 (reflecting the neutral position on the scale) and had to be as low-arousing as possible.

Since it seemed questionable whether adult norms of valence and arousal are appropriate for experiments with children, we then conducted two additional rating studies with 60 typically developing 9-year-old children and 60 adults (Bahn et al., under review). Participants judged the value of valence and arousal of each of the 48 preselected emotion terms. In contrast to the BAWL-R ratings, items in our rating studies were presented audibly in order to make the task equally feasible for children. Results showed that children’s and adults’ values were very similar (see **Table 1A**). In addition, our adult rating values for spoken stimuli were very similar to the BAWL-R rating values for written stimuli (Bahn et al., under review).

To obtain age of acquisition norms (AoA), which were not available for the full word list, two rating surveys were conducted online with 96 employees and students of two German universities (Marburg and Gießen) for the emotion terms, and 202 employees and students for the neutral words. Participants estimated on a 7-Point Likert Scale at what particular age (from the age of 2 to 8 years and older) a child most probably knows the meaning of the words. AoA values were derived from the means of all responses. Norms of concreteness for the three word sets were collected from 411 participants (employees and students) in another online rating study at the University of Marburg. Using a 7-Point Likert Scale from 1 (very abstract) to 7 (very concrete), participants were asked to assign a specific value of concreteness to each of the emotion terms and neutral words. Again, norms were derived by averaging values for each item across all participants. Values of absolute frequency (1/mil) were taken from the ChildLex corpus (Schroeder et al., 2015). This database offers frequency values for 10 million words, extracted from children’s books. With respect to the rather low frequencies of emotion terms (compared to concrete words which denote object terms) we selected neutral concrete words that were of a similar low frequency. The number of phonemes as well as norms for neighborhood density was taken from the BAWL-R.

One-way ANOVAs confirmed that: (1) the three word sets (positive, negative, and neutral) differed significantly with respect to valence, regardless of whether adult norms or children’s norms were considered. (2) Positive and negative words did not differ in their mean value of arousal, regardless of whether adult norms or children’s norms were considered. (3) Neutral words and emotion terms significantly differed with respect to arousal. This is inevitable, since emotion words always involve a higher degree of emotional activation than neutral words. (4) Neutral words and emotion terms significantly differed in their average value of concreteness: Emotion terms were less concrete than neutral words. (5) Positive and negative emotion terms did not show a difference in the mean value of concreteness. (6) The three word sets did not differ with respect to Age of Acquisition, frequency (neither for ChildLex norms, nor for CELEX norms, based on adult text corpora), morphological complexity, word length, or neighborhood density (see **Table 1B** for mean values of emotion terms and neutral words for all variables).

Next, for each word stimulus an appropriate pseudoword (*N* = 96) was generated using the software “wuggy – a multilingual pseudoword generator” by Keuleers and Brysbaert (2010). The software generates pseudowords that obey a language’s phonotactic constraints and matches all subsyllabic features and transition frequencies of the original word.

Finally, all word and pseudoword stimuli were recorded in a soundproofed booth, spoken by one female and one male trained native speaker of standard German using neutral prosody for all three word categories and pseudowords. As shown in **Table 1B**, one-way ANOVAs confirmed that there were no significant differences with regard to spoken word length (mean duration) and pitch between neutral, positive, or negative word stimuli.

### Procedure

First, all participants were informed about the study. After receiving all information and any remaining questions were answered, informed consent forms needed to be signed by all adult participants and parents. Children needed to verbally agree to their participation. Parents additionally filled in a developmental questionnaire in order to exclude any delays or disorders regarding their child’s language or cognitive development.

With 5-, 6-, and 9-year-old children, the whole procedure comprised two sessions with the following order of tasks: First session: vocabulary test and CPM, second session: LDT and VDT, whereas 12-year-olds and adults performed all tasks during one session. One break between both tasks of each session served to keep the participants attentive. Children were either tested in the laboratory, at school or kindergarten. Adults always participated in the laboratory. Children were given a small gift, adults were either monetarily rewarded or received student credit. The study was approved by the local Ethics Committee of the University of Gießen.

For the LDT, participants were seated in a quiet room with a laptop with a 15.4 inch LCD screen in front of them. Items were presented verbally via headphones. OpenSesame (Mathôt et al., 2012) was used as the controlling software. Each participant was given verbal instructions by the experimenter. Nine- and 12-year-olds as well as adults could additionally read the same instructions on the computer screen. Participants were instructed to indicate as quickly as possible via button press whether a heard string of phonemes was an existing German word (button with thumbs-up symbol) or a pseudoword without meaning (button with thumbs-down symbol). Each experimental trial (192 in total) was started by a tone (440 Hz) to capture the participant’s attention. Two hundred milliseconds later, the verbal stimulus (a neutral word, an emotion term, or a pseudoword) was played over headphones. Subsequent to the stimulus presentation, the color of the screen turned from black to green, which indicated that the participants were now allowed to press a button in response to the stimulus. The assignment of the buttons was randomized across participants to control for biases in response time due to the participants’ handedness. The button press automatically started the next trial. The semantic concept of a word vs. pseudoword as well as the meaning of the button symbols were explained to each participant using an example: “For the next several trials you will first hear a tone, and then either a real word with a meaning such as *Haus* (Eng. *house*) or a non-sense word that does not mean anything such as *mirf*. If you think that you heard a real word, press the thumbs-up button, and if you think it is a meaningless non-sense word, press the thumbs-down button”. Items were randomly presented in four blocks of 48 words and were either spoken by the female or the male speaker to avoid gender biases. Between each block a funny picture appeared on the screen in order to indicate a break. A training phase of 12 items was conducted prior to the experimental blocks to familiarize participants with the task. If the accuracy in the training was less than 75% correct responses, it was repeated once more before the experiment started.

### Data Analysis

Average accuracy and response times were calculated for each participant and item. For the analysis of accuracy, reactions from 15 5-year-olds, 21 6-year-olds, 24 9-year-olds, 24 12-year-olds, and 24 adults could be considered, which corresponded to 90% of all collected single reactions (120 participants^∗^192 words). For reaction times, from these analysable reactions outliers were excluded in a stepwise procedure: (1) only correct responses to words and pseudowords were considered for analysis (11% excluded, 89% remaining of analysable reactions). (2) Exclusion of the lowest and highest 5% of reactions that were seen as extreme outliers, e.g., due to equipment error or distraction (10% excluded, 79% remaining of all analysable reactions). (3) Exclusion of single reactions that were at the same time atypical both for a particular participant and for a particular item, i.e., above or below 2 standard deviations from the participant’s AND the item’s mean (1% excluded, 78% remaining of all analysable reactions). (4) Exclusion of participants that showed an atypical mean response time with respect to their age-mates, i.e., more than 2 standard deviations above or below the group mean (corresponds to three participants, one each for the 9-year-olds, 12-year-olds and adults, 2% reactions excluded). Thus, 76% of all analysable reactions remained for the analysis (68% of the data from the 5-year-olds, 71% from the 6-year-olds, 77% from the 9-year-olds, 78% from the 12-year-olds, and 80% of the adult data).

Using the cleaned up raw trial-by-trial data for accuracy and response time, we carried out linear (LMER) and generalized linear (GLMER) mixed effect regression analyses in the R programming environment (R Core Team, 2016) using the package lme4 (Bates et al., 2015). Prior to the analysis, all fixed predictor variables, i.e., age (between subjects, five levels: 5, 6, 9, 12 years and adult group) and valence (within-subjects, three levels: positive vs. negative vs. neutral), were effect- (i.e., deviation) coded resulting in four contrasts for age (adults = -1) and two contrasts for valence (positive = -1). All models estimated a random intercept for each participant and word-item. Type III Wald *F* Tests are reported for the LMER models (package lmerTest, Kuznetsova et al., 2016) and Type III Wald Chi-Squared Tests for the GLMER models (package car, Fox and Weisberg, 2011).

### Results

**FIGURE 1:**
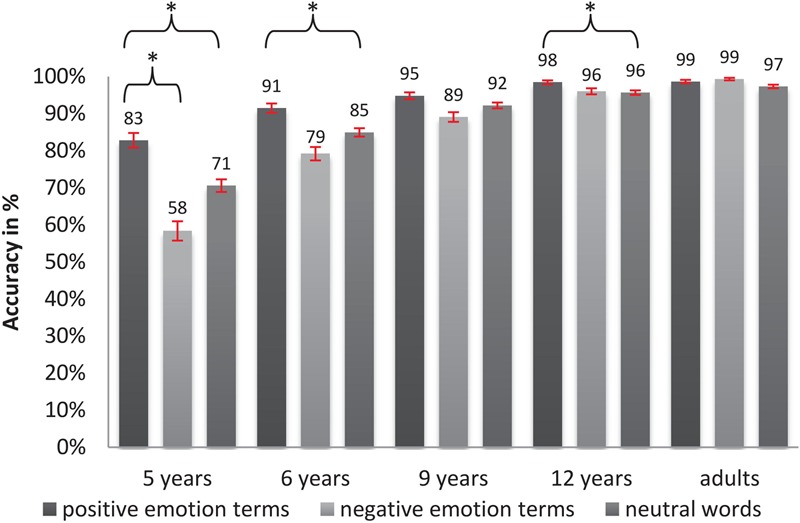
Percentages of correct responses for different word types (neutral words and positive and negative emotion terms) in LDT across age groups (error bars indicate SE and ^∗^indicate significance).

For the analysis of accuracy a generalized linear mixed-effects regression modeling approach was chosen (link = logit, fitted by Laplace approximation). The model included a random intercept for each participant and a random slope of valence for each participant. Further, we included an intercept for each word-item in the analyses. In **Table 2**, we provide the probability for a correct reaction (back transformed from the log-scale) and SE associated with each of the levels of the fixed predictors. The model revealed a significant main effect of age (*X*^2^_(4)_ = 117.57, *p* < 0.000). Estimated mean accuracy values ranged from 77% in 5-year-olds to 99% in adults. By means of a *post hoc* analysis we found that the number of correct responses significantly increased between all age groups (see all *p-* and β-values of *post hoc* comparisons in **Table 3**), except for the comparisons between the ages of 5 and 6, 6 and 9, 9 and 12, as well as between 12-year-olds and adults, where performance levels remained on a plateau. Furthermore, a significant main effect of valence (*X^2^*_(2)_ = 12.65, *p* < 0.005) and an interaction of age and valence (*X^2^*_(8)_ = 26.97, *p* < 0.001) were observed. Positive emotion terms (estimated mean accuracy of 97%), were processed with higher accuracy than negative and neutral words (*p*_positive-negative_ < 0.01, β = 6.6%, *p*_positive-neutral_ < 0.005, β = 6.7%), while negative emotion terms and neutral words did not significantly differ in accuracy (both 90% mean accuracy, *p*_negative-neutral_ = 0.999, β = 0.1%). Tukey contrasts were performed in order to further explain the influence of age on this enhanced processing of positive words compared to neutral and negative ones (interaction of age and valence, see **Figure 1**). In **Table 3**, we provide the results of *post hoc* comparisons. Results show that 5-, 6-, and 12-year-old children processed positive words with significantly higher accuracy than neutral words, while 9-year-olds and adults also showed a trend in this direction. However, a higher accuracy rate for positive words compared to negative words only reached significance in 5-year-olds (*p* < 0.01, β = 24.8%) and nearly so in 6-year-old children (*p* = 0.109, β = 6.5%).

**Table 2:** Estimated means of age and valence for LDT.

Estimated mean accuracy and mean response time with (SE)	5 years	6 years	9 years	12 years	Adults
Accuracy (positive, negative, and neutral)	77% (4.8%)	91% (2.1%)	96% (0.9%)	98% (0.4%)	99% (0.3%)
Accuracy (positive)	92% (3%)	96% (1%)	98% (0.8%)	99% (0.3%)	99% (0.3%)
Accuracy (negative)	67% (9%)	89% (4%)	96% (1%)	99% (0.5%)	100% (0.1%)
Accuracy (neutral)	73% (6%)	87% (3%)	95% (1%)	97% (0.9%)	98% (0.6%)
Response time (positive, negative, and neutral)	1049 ms (35 ms)	844 ms (30 ms)	699 ms (28 ms)	605 ms (28 ms)	319 ms (28 ms)
Response time (positive)	1015 ms (43 ms)	867 ms (36 ms)	697 ms (34 ms)	579 ms (34 ms)	300 ms (34 ms)
Response time (negative)	1005 ms (41 ms)	793 ms (35 ms)	664 ms (34 ms)	995 ms (34 ms)	301 ms (34 ms)
Response time (neutral)	1126 ms (36 ms)	871 ms (31 ms)	735 ms (29 ms)	641 ms (29 ms)	356 ms (29 ms)

**Table 3:** *Post hoc* comparisons (Tukey) on age and valence for LDT.

Accuracy age contrast	β-value	*SE*	*z*-ratio	*p*-value	Response time age contrast	β-value	*SE*	*t*-ratio	*p*-value
5–6	-13%	5%	-2.69	0.055	5-6	205 ms	45 ms	4.61	0.000
5–9	-19%	5%	-3.93	0.001	5-9	350 ms	44 ms	8.01	0.000
5–12	-21%	5%	-4.39	0.000	5-12	444 ms	44 ms	10.16	0.000
5–adults	-22%	5%	-4.51	0.000	5-adults	730 ms	44 ms	16.71	0.000
6–9	-5%	2%	-2.53	0.084	6-9	145 ms	39 ms	3.68	0.003
6–12	-8%	2%	-3.72	0.002	6-12	239 ms	39 ms	6.06	0.000
6–adults	-8%	2%	-4.01	0.001	6-adults	525 ms	39 ms	13.33	0.000
9–12	-2%	0.9%	-2.45	0.102	9-12	94 ms	38 ms	2.43	0.115
9–adults	-3%	0.9%	-3.21	0.012	9-adults	380 ms	38 ms	9.88	0.000
12–adults	-0.7%	0.4%	-1.46	0.588	12-adults	286 ms	38 ms	7.45	0.000

**Accuracy valence contrast**	**β-value**	***SE***	***z*-ratio**	***p*-value**	**Response time valence contrast**	**β-value**	***SE***	***t*-ratio**	***p*-value**

5 years positive-negative	25%	8%	3.05	0.006	5 years positive-negative	9 ms	34 ms	0.29	0.955
5 years positive-neutral	18%	6%	3.27	0.003	5 years positive-neutral	-111 ms	34 ms	-3.32	0.003
5 years negative-neutral	6%	8%	-0.78	0.714	5 years negative-neutral	-121 ms	34 ms	-3.58	0.001
6 years positive-negative	7%	3%	2.01	0.110	6 years positive-negative	73 ms	29 ms	-2.57	0.030
6 years positive-neutral	8%	3%	2.78	0.015	6 years positive-neutral	-5 ms	29 ms	-0.17	0.985
6 years negative-neutral	2%	4%	0.52	0.864	6 years negative-neutral	-78 ms	30 ms	-2.58	0.029
9 years positive-negative	2%	1%	1.29	0.402	9 years positive-negative	33 ms	28 ms	1.20	0.454
9 years positive-neutral	3%	1%	2.12	0.087	9 years positive-neutral	-37 ms	28 ms	-1.32	0.384
9 years negative-neutral	1%	2%	0.81	0.698	9 years negative-neutral	-71 ms	30 ms	-2.38	0.049
12 years positive-negative	0.5%	0.5%	1.02	0.566	12 years positive-negative	-16 ms	27 ms	-0.60	0.821
12 years positive-neutral	2%	0.9%	2.64	0.023	12 years positive-neutral	-63 ms	28 ms	-2.23	0.069
12 years negative-neutral	2%	0.9%	3.03	0.105	12 years negative-neutral	-46 ms	30 ms	-1.57	0.264
Adults positive-negative	-0.4%	0.3%	-1.44	0.321	Adults positive-negative	-1 ms	27 ms	-0.04	0.999
Adults positive-neutral	1%	0.6%	2.13	0.083	Adults positive-neutral	-56 ms	28 ms	-2.01	0.113
Adults negative-neutral	2%	0.6%	2.93	0.010	Adults negative-neutral	-55 ms	29 ms	-1.87	0.150

**FIGURE 2:**
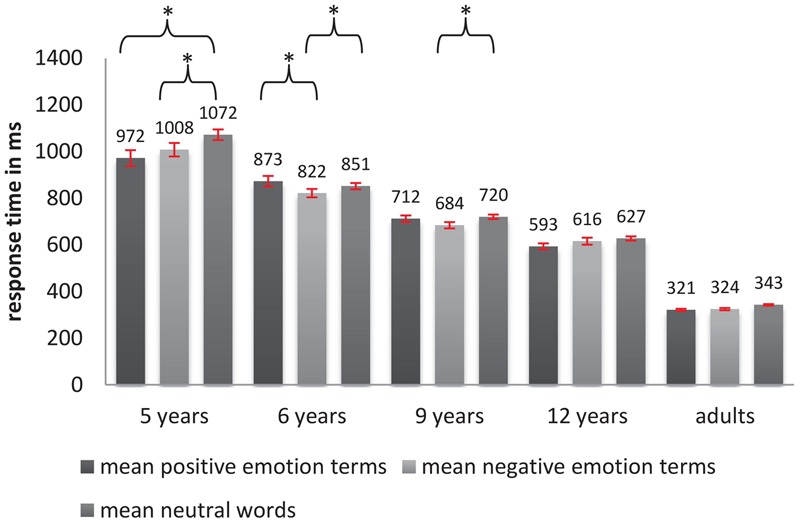
Average response times for different word types (neutral words and positive and negative emotion terms) in LDT across age groups (error bars indicate SE and ^∗^indicate significance).

Turning to the analysis of response times, we implemented a linear mixed-effects regression approach (fitted by Restricted Maximum Likelihood, with Satterthwaite approximations to degrees of freedom). Here, we estimated a random intercept for each participant and a random slope of valence for each participant. Further, we included a random intercept for each word-item. Age and valence were included as fixed predictors as described above. Since neutral words and emotion terms (positive vs. negative) significantly differed with respect to their mean concreteness value, concreteness was added as a continuous covariate (grand-mean centered) to the model in order to control for its potential influence. Although a main effect of concreteness could be observed [*F*_(1,92)_= 3.82, *p* < 0.005], there were no significant interactions between concreteness and the other predictors (all *p*s > 0.05). **Figure 2** shows the mean accuracies for each age group and each word category. A main effect of age [*F*_(4,99)_ = 83.37, *p* < 0.000] was significant, showing that average response times (averaged over positive, negative, and neutral words) ranging from 1047 ms in 5-year-olds to 341 ms in adults significantly decreased as a function of age (*p*_6and9_ _years_ < 0.005, all other *p*-values < 0.0001) except for between the ages of 9 and 12 (*p* = 0.115). Further, results showed a significant main effect of valence [*F*_(2,96)_ = 4.66, *p* < 0.01]: Negative words were processed slightly faster than positive words (estimated mean response time of negative words 672 ms and of positive words 691 ms) with neutral words showing the slowest responses (746 ms). However, as can be seen in **Table 3**, Tukey-*post hoc* analyses revealed that only negative words were processed significantly (and positive words almost significantly) faster than neutral words (*p*_negative-neutral_ < 0.001, β = -74 ms, *p*_positive-neutral_ = 0.06, β = -55 ms), whereas negative and positive words did not differ significantly in processing speed. Furthermore, an interaction of valence and age [*F*_(8,139)_ = 2.65, *p* < 0.05, see **Figure 2**] suggests that the valence-related differences in response time are modulated by the participants’ age: Negative words were processed faster compared to neutral words in 5-, 6-, and 9-year-old children, positive words were processed faster than neutral words in 5-year-old children, and the difference between negative and positive words (with negative words being processed faster than positive words) only reached significance in the 6-year-old children.

### Discussion

As expected, an age-dependent improvement in LDT performance (higher accuracy and faster responses) was observed, suggesting that in general, children become more mature in accessing information from the mental lexicon as they grow older. Regarding developmental trajectories, patterns for accuracy and response times turned out to be somewhat different: With respect to accuracy, improvement was most pronounced between the two youngest groups (from 77% in 5-year-olds to 91% in 6-year-olds). After this early developmental boost, there were no other significant differences between two adjacent age groups, which points to a continuous, i.e., a slow but steady growth in accuracy over a long time period beginning at age 6. For reaction time, no such early developmental boost was observed. The results rather point to a continuous acceleration of processing speed over middle childhood with a developmental plateau between 9 and 12 years of age.

With respect to valence, we found a positivity advantage in accuracy which is in line with previous findings (Kuchinke et al., 2005, 2007; Kanske and Kotz, 2007; Hofmann et al., 2009; Scott et al., 2009, 2014; Palazova et al., 2011; Bayer and Schacht, 2014; Ferré and Sanchez-Casas, 2014; Kuperman et al., 2014; Ponari et al., 2015; Yao et al., 2016). Positive words were not only processed more correctly compared to negative words, but also more correctly than neutral words. Furthermore, the effect turned out to be modulated by age: Only 5- and 6-year-olds were more accurate in identifying positive words than negative words. Possible explanations for this early and gradually decreasing positivity advantage will be discussed in the general discussion. For response times, however, a slight but significant main effect of valence in the reversed direction (with negative words being processed faster than positive words) was found, which was solely caused by one age group – the 6-year-old children. This finding contrasts with the faster processing of positive as opposed to negative words, found in our children aged 5 and 12 and in the 12-year-old children reported by Sylvester et al. (2016).

## Experiment 2 – Emotional Categorization (VDT)

### Participants

Experiment 2 was performed by the same children and adults who already participated in experiment 1 (see section “Participants” of Experiment 1 for a detailed description of participants). Three percent of the participants (two 5-year-olds and two 6-year-olds) needed to be excluded due to achieving a mean accuracy of less than 60%.

### Stimuli

In the VDT, we used a subset of the stimuli from Experiment 1: 24 positive and 24 negative German emotion terms. See Section “Stimuli” of Experiment 1 for a detailed description of selection criteria and the process of stimulus construction.

### Procedure

The procedure of the VDT was identical to the LDT except for the task-relevant instruction. As in the LDT, participants were informed that they will hear a tone followed by a word at which point they had to indicate whether that word carried a positive or negative meaning as quickly and accurately as possible. This time, a sun symbol indicated a positive word meaning, and a raincloud indicated a negative one. The meaning of “positive” and “negative” was further explained to all children using phrases such as “positive means something good, nice, or pleasant. For example, the word *love* is positive. When you think a word is positive, press the button with the sun, when you think it has a negative meaning, press the button with the raincloud.” Again, 12 items were presented to train participants on the task before the first experimental trial started, and all items were presented in a randomized order. Since only 48 trials (24 positive and 24 negative items) were presented in this task, two blocks with one small break in between were sufficient.

### Data Analysis

As for the LDT, average accuracy and response times were calculated for each participant and item. Twenty-two participants in each of the 5- and 6-year-old group as well as 24 participants in each of the 9-year-old, 12-year-old, and adult group were left for the analysis of accuracy, which corresponds to 97% of all collected single reactions (120 participants^∗^48 items). Compared to the LDT, one additional exclusion criterion (1.1) was added to the data reduction process: The VDT responses for words for which a child gave an incorrect response in the LDT were excluded from the data analysis, assuming that one cannot adequately determine a word’s valence without first knowing that it is a word at all. Based on five steps (the same as for LDT except for 1.1) the following proportions of single reactions were excluded from the response time data: (1) only correct responses from the VDT were considered for analysis (11% excluded, 89% remaining of all analysable reactions). (1.1) After exclusion of words with incorrect responses in the LDT from the VDT data (9%) 80% of all analysable reactions remained for the analysis. (2) Exclusion of the lowest and highest 5% of reactions that were seen as extreme outliers, e.g., due to equipment error or distraction (9% excluded, 71% remaining of all analysable reactions). (3) Exclusion of single reactions that were at the same time atypical for both a particular participant AND for a particular item, i.e., above or below 2 standard deviations from the participant’s and item’s mean (2% excluded, 69% remaining of all analysable reactions). (4) Exclusion of participants that showed an atypical mean response time with respect to their age-mates again using the rule of 2 standard deviations from the mean (corresponds to five participants, one each for every age group, 3% reactions excluded). In total, 66% of all analysable reactions could be considered for reaction time analysis (42% of the data from the 5-year-olds, 61% from the 6-year-olds, 73% from the 9-year-olds, 76% from the 12-year-olds, and 78% of the adult data).

We carried out LMER and GLMER mixed effect regression analyses in the R programming environment (R Core Team, 2016) as described above (see Experiment 1: Data Analysis).

### Results

**FIGURE 3:**
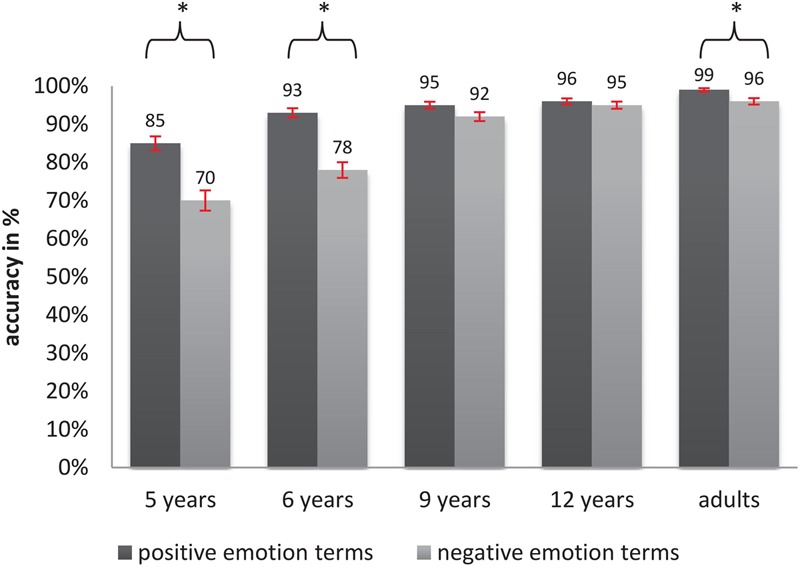
Percentages of correct responses for positive and negative emotion terms in VDT across age groups (error bars indicate SE and ^∗^indicate significance).

For the analysis of accuracy, we once again used a generalized linear mixed-effects regression modeling approach (link = logit, fitted by Laplace approximation). The model included a random intercept for each participant and a random intercept for each word. Age and valence were included as fixed predictors as described above. **Table 4** provides the probability for a correct reaction (back transformed from the log-scale) and *SE* associated with each of the levels of the fixed predictors. The model revealed that age significantly influenced the number of correct responses (main effect of age, [*X^2^*_(4)_ = 122.97, *p* < 0.000]. The mean probability for giving a correct response ranged from 79% in 5-year-olds to 98% in adults. Results from *post hoc* comparisons (**Table 5**), confirmed a significant increase of correct responses between 5-year olds, and 9-year-olds, 12-year-olds, and adults (all *p*-values < 0.0001) as well as between 6-year-olds and all older age groups (all *p*-values < 0.001). Performance remained on a plateau between 5 and 6 years of age, and between 9 years of age until adulthood. A significant main effect of valence [*X^2^*_(1)_ = 21.14, *p* < 0.000] showed that the mean number of correct responses for positive words (95%, *SE* = 0.8%) was higher than for negative words (88%, *SE* = 1.5%, contrast negative–positive, *p* < 0.0001, β = -7.2%). Additionally, a significant interaction of valence and age was detected [*X^2^*_(4)_ = 12.39, *p* < 0.05]. **Figure 3** and **Table 5** show that accuracy was higher for positive words in 5- and 6-year-old children as well as in adults (*p*_5__years_ < 0.001, contrast negative–positive β = -15.8%, *p*_6__years_ < 0.0001, contrast negative–positive β = -14.3%, *p*_adults_ < 0.001, contrast negative–positive β = -2%). Nine-year-olds showed a trend toward an enhanced (more correct) processing of positive words compared to negative ones (*p* = 0.06, contrast negative–positive β = -7.2%), while no valence-specific difference appeared in 12-year-olds.

**Table 4:** Estimated means of age and valence for VDT.

Estimated mean accuracy and mean response time with (SE)	5 years	6 years	9 years	12 years	Adults
Accuracy (positive and negative)	79% (3%)	87% (2%)	96% (1%)	97% (1%)	98% (0.4%)
Accuracy (positive)	87% (3%)	94% (1%)	97% (1%)	97% (1%)	99% (0.2%)
Accuracy (negative)	71% (5%)	80% (4%)	94% (1%)	96% (1%)	97% (1%)
Response time (positive and negative)	1033 ms	1003 ms	890 ms	770 ms	325 ms
Response time (positive)	944 ms	927 ms	864 ms	753 ms	322 ms
Response time (negative)	1122 ms	1078 ms	917 ms	788 ms	329 ms

**Table 5:** *Post hoc* comparisons (Tukey) on age and valence for VDT.

Accuracy age contrast	β-value	SE	*z*-ratio	*p*-value	Response time age contrast	β-value	*SE*	*t*-ratio	*p*-value
5–6	-8%	3%	-2.45	0.102	5–6	30 ms	69 ms	0.44	0.992
5–9	-17%	3%	-5.38	0.000	5–9	143 ms	67 ms	2.12	0.218
5–12	-18%	3%	-5.82	0.000	5–12	263 ms	67 ms	3.92	0.002
5–adults	-19%	3%	-6.19	0.000	5–adults	708 ms	67 ms	10.55	0.000
6–9	-8%	2%	-3.94	0.001	6–9	112 ms	66 ms	1.72	0.430
6–12	-10%	2%	-4.63	0.000	6–12	232 ms	65 ms	3.55	0.005
6–adults	-11%	2%	-5.22	0.000	6–adults	678 ms	65 ms	10.36	0.000
9–12	-1%	1%	-1.31	0.684	9–12	120 ms	64 ms	1.90	0.330
9–adults	-2%	0.9%	-2.63	0.065	9–adults	565 ms	64 ms	8.90	0.000
12–adults	-1%	0.7%	-1.50	0.560	12–adults	445 ms	63 ms	7.02	0.000

**Accuracy time valence contrast**	**β-value**	***SE***	***z*-ratio**	***p*-value**	**Response time valence contrast**	**β-value**	***SE***	***t*-ratio**	***p*-value**

5 years positive–negative	-16%	4%	-3.50	0.001	5 years positive–negative	178 ms	54 ms	3.31	0.001
6 years positive–negative	-14%	3%	-4.29	0.000	6 years positive–negative	151 ms	47 ms	3.21	0.002
9 years positive–negative	-3%	1%	-1.86	0.064	9 years positive–negative	53 ms	44 ms	1.21	0.232
12 years positive–negative	-1%	1%	-1.05	0.292	12 years positive–negative	36 ms	43 ms	0.82	0.414
Adults positive–negative	-2%	0.8%	-3.19	0.001	Adults positive–negative	7 ms	43 ms	0.17	0.868

**FIGURE 4:**
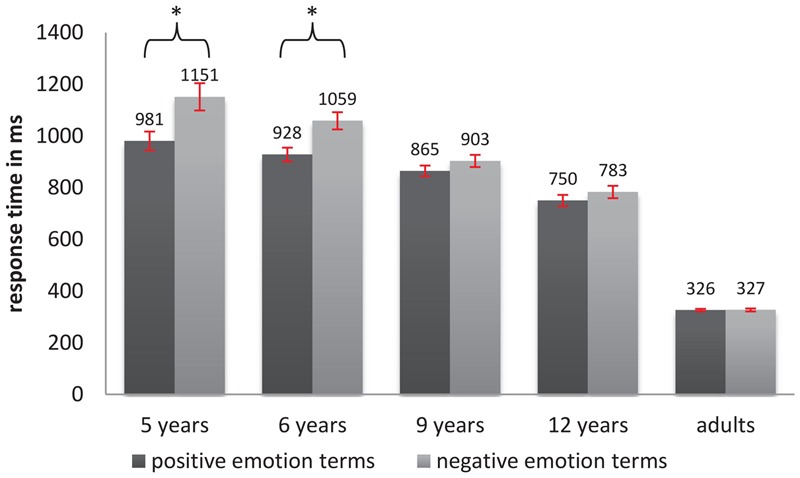
Average response times for positive and negative emotion terms in VDT across age groups (error bars indicate SE and ^∗^indicate significance).

Results with respect to response times revealed a comparable pattern. Here, we implemented a linear mixed-effects regression approach (fitted by Restricted Maximum Likelihood, with Satterthwaite approximations to degrees of freedom) estimating a random intercept for each participant, a random slope of valence for each participant and a random intercept for each word-item. Age and valence were included as fixed predictors as described above. All estimated average response times are shown in **Table 4**. Average response times ranged from 1033 ms in 5-year-olds to 325 ms in adults. A significant main effect of age was detected [*F*_(4,99)_ = 39.09, *p* < 0.000]. *Post hoc* comparisons (values are displayed in **Table 5**) confirmed a significant decrease of response times between every child group and adults, as well as between 5- and 12-year-olds (*p* < 0.005, β = 263 ms, *SE* = 67 ms), and between 6-year-olds and 12-year-olds (*p* < 0.01, β = 232 ms, *SE* = 65 ms). No increase in processing speed could be observed between the ages of 5 and 9 years, and between 9 and 12 years. Regarding valence, the analysis revealed a significant main effect [*F*_(1,50)_ = 6.40, *p* < 0.01] indicating that positive words were processed more quickly than negative words overall (*p*_negative-positive_ < 0.01, β = 85 ms, *SE* = 33 ms). An interaction of valence and age also reached significance [*F*_(4,77)_ = 3.99, *p* < 0.01]. As shown in **Figure 4** and **Table 5**: the increase in processing speed for positive words only appeared in the two youngest age groups, i.e., in 5- and 6-year-old children (*p*_5__years_ < 0.005, contrast negative–positive β = 178 ms, *SE* = 54 ms, *p*_6__years_ < 0.005, contrast negative–positive β = 151 ms, *SE* = 47 ms).

### Discussion

In accordance with our hypothesis, accuracy rates and processing speed in VDT increased with the age of the participants. Furthermore, the developmental pattern of this age-related improvement in emotion term processing seems to differ with respect to the measure of performance. Regarding accuracy, the biggest developmental change seemed to occur between the ages of 6 and 9. During this time period children become proficient in correctly categorizing emotion terms along their valence, which finally results in an adult-like accuracy at the age of 9. However, response times of children remain slower compared to those of adults throughout the entire range of children’s ages that were tested. Furthermore, the increase of processing speed occurs steadily during a long developmental phase from 5 to 12 years of age.

Both measures of performance showed the predicted modulation by the stimulus’ valence and the participants’ age: results point to an early advantage (faster and more accurate processing in 5- and 6-year olds) of positive words compared to negative words. For accuracy, we additionally found a positivity bias in adults and a trend toward the same for 9-year-old children. However, given that adults made only a few errors in VDT overall, one more single wrong response to a negative word (compared to reactions to positive words) might have influenced the appearance of the valence effect more strongly than would have been the case for children. For this reason, our results point toward an overall continuous decrease up to an absence of the positivity bias in older children and adults. This, however, does not fully converge with the results of Sylvester et al. (2016), who found an increase of processing speed in VDT for positive words in children aged 7, 9, and 12 years and well as with several other studies that showed a positivity advantage in adults (e.g., Feyereisen et al., 1986; Hofmann et al., 2009; Bayer and Schacht, 2014; Goh et al., 2016). Possible explanations for these heterogeneous findings will be discussed in the following section in connection with the LDT results.

## Summary and General Discussion

The aim of the present study was to detect developmental changes in emotion word processing. Performance in two psycholinguistic tasks (LDT and VDT) was compared and analyzed regarding possible influences of the two factors of age (children aged 5, 6, 9, 12 and adults) and valence (neutral words vs. positive emotion terms vs. negative emotion terms). To our knowledge, this was the first study that aimed at detecting similarities or differences in emotion word processing between children of different age groups as well as between children and adults. Furthermore, the presented sets of emotion terms and neutral words were carefully controlled for valence, arousal, age of acquisition, concreteness, frequency and a number of linguistic variables to ensure that valence and age effects should not be weakened by confounding emotional or linguistic factors.

Briefly, our results clearly demonstrate a general improvement of word processing with increasing age. In addition to this expected general improvement with age, we were able to uncover developmental trajectories. Depending on task and outcome measure, we found characteristic patterns of development over time. Most importantly, the present study demonstrated a shift in the processing of positive and negative words in the course of development: While young children showed a better performance for positive words, this preference disappeared with increasing age. Possible explanations for the age- and valence-related findings will be discussed below.

### Age Effects

Age effects were stable across outcome measures and task. As expected, performance in both tasks became significantly better (higher accuracy and faster responses) with increasing age, suggesting that mental representations of emotion terms become better accessible with age. Furthermore, age-related trajectories were characterized by both a continuous improvement of processing abilities and by developmental boosts followed by plateaus, depending on the outcome measure (accuracy or response time) as well as on the task. For LDT and VDT, children reached an adult-like performance level in accuracy, whereas response times continued to improve until adulthood. Five- and six-year-old children showed severe difficulties in the LDT, where they had to discriminate between words and pseudowords. Their performance in this task should therefore be interpreted with caution. In 5-year-olds 38%, and in 6-year-olds 13% of the children had to be excluded because of accuracy scores below 60%. Their lower performance levels might have appeared for different reasons: (1) Although the selected positive, negative, and neutral words had an average age of acquisition value of less than six years of age (see **Table 1B**), some of the young children may not have yet been familiar with the word form and/or the meaning of individual words. This might have resulted in high error rates due to missing or incomplete mental representations of the presented words, as well as due to problems in quickly accessing lexical units from the mental lexicon in order to discriminate between words vs. pseudowords. As participants become older, their semantic network becomes more elaborated and interconnected (He and Arunachalam, 2017) and therefore lexical units can be accessed faster. (2) The need to categorize information as positive or negative is highly relevant for adaptive processes and appears more frequently in daily life compared to the need to distinguish words from pseudowords. In this regard, the VDT might have been a more intuitive task than the LDT, which is perhaps why differences in performance between the two tasks were especially pronounced in young children.

### Valence Effects

Children at the age of 5 and 6 showed a stable positivity advantage in three of the four different outcome measures (LDT: positivity advantage in accuracy, VDT: positivity advantage in accuracy and response times). Responses to positive words in these two age groups were faster and more accurate than to negative words or neutral words (in LDT). This result matches the findings by Peréz-Edgar and Fox (2007) as well as by Sylvester et al. (2016). A possible explanation may be found in the informational density hypothesis as suggested by Unkelbach et al. (2008) and Sylvester et al. (2016, see Introduction): the processing of negative words requires more cognitive resources, since children’s semantic representation of positive words might be more densely clustered and more elaborated than that of negative words. Furthermore, emotions are more diverse in the negative domain. Thus, the emotion vocabulary contains more lexical units for expressing negative than for positive emotions. It might therefore be easier for young children to express positive events through words, since they do not need so many different words for expressing positive emotions. Neshat-Doost et al. (1999) analyzed the productions of positive and negative emotional words from primary and secondary school children in a free word production task. The children were asked to name (1) as many words that describe a positive or negative feeling and (2) things that can evoke positive or negative feelings. The results showed that the number of words differed with respect to valence: While primary and secondary school children produced the same number of positive words, younger children produced significantly fewer negative words compared to the older children. This finding indicates that the positive emotion vocabulary is acquired earlier in the course of language development than the vocabulary for negative words. Another reason for the enhanced processing of positive words might be that the younger children were more familiar with, i.e., had been more exposed to positive words compared to the negative ones. Even though we controlled the words for estimated age of acquisition and frequency, only values for written frequencies, but not for oral frequencies were available. It cannot be ruled out that children might have heard positive words more often in their first years of life compared to negative words. Dodds et al. (2014) obtained adults’ valence ratings for the 10.000 most frequently used words of 10 languages. Results revealed a general positivity bias across languages.

Considering the large body of studies on non-verbal emotion processing with infants and children that strongly points toward an enhanced processing of negative cues compared to positive ones (e.g., Vaish et al., 2008), the appearance of the opposite pattern for linguistic stimuli in the present study, i.e., the positivity bias in emotion term processing of 5- and 6-year-olds, is rather surprising. It may be that the direction of valence effects in emotion processing differs with respect to modality. The evaluation of non-verbal emotional information may more strongly serve evolutionary adaptive functions and would therefore more likely lead to a negativity bias compared to the evaluation of verbal emotional cues, because the ability to interpret non-verbal cues develops long before specific lexical units for emotions are acquired. In contrast, the processing of verbally conveyed emotional information may result in an advantage for positive information, caused by a higher exposure frequency which would then carry more weight than evolutionary adaptive functions. In sum, the early positivity advantage observed in our data might be due to a higher experience with positive emotion terms and/or due to their dense representation in the mental lexicon.

As mentioned in the introduction, Estes and Verges (2008) state that valence effects are modulated by the applied task. In line with these authors, we found a similar effect in children, since the valence effect was stronger in the VDT, (revealed in accuracy and response time), for which the stimuli’s valence is more relevant compared to the LDT, for which no semantic information must be retrieved from memory. For the latter, the valence effect only appeared in accuracy. Hence, the more important valence is for the task, the higher is the impact of valence on children’s emotion term processing.

Although the results of the present study confirmed the expected valence effect (in the form of a positivity bias), as well as a modulation of this effect by the factor age, the (almost complete) absence of the positivity bias in older children and adults contrasts with a large number of the previously reported findings (e.g., Estes and Verges, 2008; Hofmann et al., 2009; Bayer and Schacht, 2014; Goh et al., 2016). One possible reason why older children and adults did not show a valence effect with respect to accuracy might be that they were limited by ceiling effects in both tasks. Ceiling effects could not be completely prevented, because the item set had to be appropriate for the participants of all five age groups. However, ceiling effects for accuracy alone cannot explain the absence of a valence effect in older participants, since the same pattern (decreasing positivity bias) was also found for response times. Older children, who did not show more correct responses to positive words anymore, nevertheless did not reach an adult-like level of processing speed, which indicates that the task was still challenging for them. Similar to a ceiling effect in accuracy, a possible processing advantage of positive over negative words in response times might have been concealed by a floor effect. For methodological reasons, participants were only allowed to respond to each verbal stimulus when it was completely presented. This was done to avoid provoking too many incorrect responses (especially in the children) due to premature responses to pseudowords that differ from real words in their last syllable. However, mature speakers would probably have shown faster responses for either positive or negative words in the two applied word processing tasks had they not been slowed down artificially. In other words, the critical time slot in which a positivity bias would have appeared was probably not considered in the analysis.

It is also important to mention that the comparability of the results of previous studies and those of the present investigation is reduced for several reasons: (1) we controlled the stimuli for arousal, while other studies that found valence effects in adults did not report doing so (e.g., Feyereisen et al., 1986; Palazova et al., 2013). (2) The majority of studies presented written words, while we used audibly presented words in order to make the task feasible for young children with no or developing reading abilities. It is possible that visually presented emotional words might be perceived differently from their spoken equivalents (Feyereisen et al., 1986). (3) Other studies used a mixture of concrete and abstract affective words and emotion terms (three types of words that are likely to differ with respect to concreteness) while in the present study an exclusive set of emotion terms was used with an equal mean value of concreteness for positive and negative words. Since research has shown that concreteness impacts on word processing, it could be that concreteness and valence effects occur in parallel, interact with each other and impact VDT and LDT performance if one does not control for this factor.

## Conclusion

The present study showed that children’s processing of emotion terms, as investigated by two word processing tasks (LDT and VDT), improves during childhood. While both tasks were difficult for young children (age 5 and 6), children at the age of 9 and 12 had acquired well-specified semantic representations of emotion terms, as reflected by almost adult-like error rates in both tasks. Regarding processing speed, development continued until adulthood. The focus of the present study was on the effect of valence in emotion term processing. The results demonstrated a clear positivity advantage that turned out to be age- as well as task-dependent: First, preferential processing of positive over negative terms was characteristic for young children, but decreased with age. Second, the valence effect was more pronounced in the emotional categorization task, where access to a word’s valence is strongly task-relevant.

Our findings demonstrate a positivity bias in children for emotion terms exclusively. Future studies should investigate children’s word processing using matched sets of emotion terms, and of affective words with different levels of concreteness. In addition, words were presented in isolation in the present study, and not embedded in a linguistic context. Therefore, no conclusions can be drawn about children’s perception of emotion terms in natural communication. Recent studies point toward a strong influence of contextual information on emotion processing (Rohr and Rahman, 2015; Suess et al., 2015). Future research should therefore move beyond the single word level in order to further explore the reasons for the early processing preference for positive words and its subsequent decrease as children grow older.

## Ethics Statement

This study was carried out in accordance with the recommendations of “Lokale Ethikkommission des Fachbereichs 06 Justus-Liebig-Universität Gießen” with written informed consent from all subjects. All subjects gave written informed consent in accordance with the Declaration of Helsinki. The protocol was approved by the “Lokale Ethikkommission des Fachbereichs 06 Justus-Liebig-Universität Gießen.

## Author Contributions

Acquisition, analysis, and interpretation of data for the work: DB, MV, JG, GS, and CK. Drafted the work and revised it critically for important intellectual content: DB, MV, JG, GS, and CK. Final approval of the version to be published: DB, MV, JG, GS, and CK. Agreement to be accountable for all aspects of the work in ensuring that questions related to the accuracy or integrity of any part of the work are appropriately investigated and resolved: DB, MV, JG, GS, and CK.

## Conflict of Interest Statement

The authors declare that the research was conducted in the absence of any commercial or financial relationships that could be construed as a potential conflict of interest.
